# Stormorken Syndrome Caused by a p.R304W *STIM1* Mutation: The First Italian Patient and a Review of the Literature

**DOI:** 10.3389/fneur.2018.00859

**Published:** 2018-10-15

**Authors:** Oscar Borsani, Daniela Piga, Stefania Costa, Alessandra Govoni, Francesca Magri, Andrea Artoni, Claudia M. Cinnante, Gigliola Fagiolari, Patrizia Ciscato, Maurizio Moggio, Nereo Bresolin, Giacomo P. Comi, Stefania Corti

**Affiliations:** ^1^Neurology Unit, Fondazione IRCCS Ca' Granda Ospedale Maggiore Policlinico, Milan, Italy; ^2^A. Bianchi Bonomi Hemophilia and Thrombosis Center, Fondazione IRCCS Ca' Granda Ospedale Maggiore Policlinico, Milan, Italy; ^3^Neuroradiology Unit, Fondazione IRCCS Ca' Granda Ospedale Maggiore Policlinico, Milan, Italy; ^4^Neuromuscular and Rare Diseases Unit, Department of Neuroscience, Fondazione IRCCS Ca' Granda, Ospedale Maggiore Policlinico, Milan, Italy; ^5^Neuroscience Section, Department of Pathophysiology and Transplantation, Dino Ferrari Centre, University of Milan, Milan, Italy

**Keywords:** stormorken syndrome, STIM1, tubular aggregate myopathy, muscle, myopathy

## Abstract

Stormorken syndrome is a rare autosomal dominant disease that is characterized by a complex phenotype that includes tubular aggregate myopathy (TAM), bleeding diathesis, hyposplenism, mild hypocalcemia and additional features, such as miosis and a mild intellectual disability (dyslexia). Stormorken syndrome is caused by autosomal dominant mutations in the *STIM1* gene, which encodes an endoplasmic reticulum Ca^2+^ sensor. Here, we describe the clinical and molecular aspects of a 21-year-old Italian female with Stormorken syndrome. The *STIM1* gene sequence identified a c.910C > T transition in a STIM1 allele (p.R304W). The p.R304W mutation is a common mutation that is responsible for Stormorken syndrome and is hypothesized to cause a gain of function action associated with a rise in Ca^2+^ levels. A review of published *STIM1* mutations (*n* = 50) and reported Stormorken patients (*n* = 11) indicated a genotype-phenotype correlation with mutations in a coiled coil cytoplasmic domain associated with complete Stormorken syndrome, and other pathological variants outside this region were more often linked to an incomplete phenotype. Our study describes the first Italian patient with Stormorken syndrome, contributes to the genotype/phenotype correlation and highlights the possibility of directly investigating the p.R304W mutation in the presence of a typical phenotype.

**Highlights**

- Stormorken syndrome is a rare autosomal dominant disease.
- Stormoken syndrome is caused by autosomal dominant mutations in the *STIM1* gene.
- We present the features of a 21-year-old Italian female with Stormorken syndrome.
- Our review of published *STIM1* mutations suggests a genotype-phenotype correlation.
- The p.R304W mutation should be investigated in the presence of a typical phenotype.

## Introduction

Stormorken syndrome is a rare disorder (OMIM: #185070) with a complex phenotype comprising muscle fatigue associated with tubular aggregate myopathy (TAM), asplenia, a bleeding diathesis associated with thrombocytopenia and/or thrombocytopathy, miosis, and additional features, including ichthyosis, short stature, a minimal cognitive impairment (dyslexia) and migraines (1–6).

TAM is a rare disorder in which tubular aggregates are the main pathological hallmarks in muscle tissue (7). Tubular aggregates may also be found in congenital myasthenic syndromes (8) and in other hereditary myopathies as an accompanying feature (9). Tubular aggregates are composed of tubules derived from the sarcoplasmic reticulum oriented in regular arrays (10). A “pure” form of TAM, which is clinically characterized by cramps, myalgias, muscle stiffness and, eventually, proximal muscle weakness, has been described. TAM may be sporadic or inherited as an autosomal dominant trait (9).

Heterozygous dominant mutations in the *STIM1* gene (chromosome 11p15) have been determined to be causative of Stormorken syndrome (6). The *STIM1* gene encodes the major Ca^2+^ sensor of the endoplasmic reticulum (ER), called “stromal interaction molecule 1” (STIM1) (11, 12).

STIM1 is a conserved protein in which the N-terminal (luminal) region is separated from the C-terminal (cytoplasmic) domain by a single transmembrane domain. The C-terminal domain harbors three coiled coil (CC) domains, and the N-terminal region harbors two EF-hand domains that act as calcium sensors within the ER. Depletion of calcium from the endoplasmic reticulum causes the dissociation of Ca^2+^ from the STIM1 luminal domain, which in turn, induces the generation of higher-order STIM1 oligomers. These changes in the STIM1 conformation were proposed to lead to its migration toward the so-called “puncta regions,” which are discrete cell sites originating from the endoplasmic reticulum-plasma membrane (ER–PM) apposition. In these sites, STIM1 oligomers interact with ORAI1, a cytoplasmic membrane Ca^2+^ release-activated Ca^2+^ (CRAC) channel, causing the ORAI1 channel to open, as well as stimulating store-operated calcium entry (SOCE) (11, 12). STIM1 protein oligomerization may be constitutively activated by STIM1 gene mutations (such as R304W and L251S) located in the region encoding the cytosolic domain. These events lead to STIM1 puncta development and Ca^2+^ influx through CRAC channels. These mutations lead to a STIM1 gain of function and can mimic the activated conformations of STIM1 (1, 12, 13). In addition to STIM1, TAM may be caused by mutations in ORAI1 (1, 14).

The most common STIM1 mutation (c.910C>T, p.R304W) described in subjects affected by classic (complete) Stormorken syndrome involves the first CC domain (1, 2, 6). However, several heterozygous mutations have been described in the EF-hand domain of *STIM1*, leading to incomplete phenotypes, such as non-syndromic tubular aggregate myopathies (TAM) with miosis and York platelet syndrome (YPS) (15). There is marked diversity in the neuromuscular features of subjects with mutations in the EF-hand vs. those with mutations in the CC domain. In particular, subjects with a mutation in the EF-hand have an earlier onset of neuromuscular symptoms and higher prevalence of myalgia, contractures, ophthalmoplegia, and progressive weakness than those with mutations in the CC domain (4). Finally, recessive mutations in STIM1 have been described to be associated with immunodeficiency and Kaposi's sarcoma (16–18). Taken together, these data suggest a possible genotype–phenotype correlation associated with the site of the mutation.

Here, we describe a novel case of a 21-year-old Italian patient with the p.R304W *STIM1* mutation who presented with the classic Stormorken phenotype.

## Materials and methods

The subject of this observational study was evaluated at the IRCCS Foundation Ca' Granda Maggiore Hospital was performed according to the Declaration of Helsinki. Written informed consent was obtained from the participant for the publication of this case report. A complete neurological examination was performed, and signs of Stormorken syndrome were examined in the proband. The study was extended to the parents.

The blood tests performed included evaluation of the full blood count, liver function tests, inflammatory markers, serum electrolytes, markers of renal function, creatine phosphokinase (CK), thyroid stimulating hormone (TSH), parathyroid hormone (PTH), and coagulation.

To assess whether clinical and biochemical features were associated with the involvement of the nerves and muscles, neurophysiological studies were conducted, including a nerve conduction study (NCS) and needle electromyography (EMG).

A standard brain MRI and muscle MRI were performed with 1.5 Tesla equipment (Philips Achieva, Eindhoven, the Netherlands). Non-contrast axial images were generated using T1-TSE (turbo spin echo) sequences to detect fatty replacement. Short tau inversion recovery (STIR) sequences were acquired on the axial plane to determine muscle edema.

A biopsy of the patient's left biceps muscle was performed, and 10 μm cryostatic cross sections were processed according to standard histological/histochemical techniques (Gomori Trichrome and Hematoxylin and eosin stain) (19). Enzymatic activity was demonstrated by ATPASE (9.4, 4.6, 4.3), COX, succinic dehydrogenase (SDH) and double staining for COX and SDH, acid phosphatase, NADH, Oil Red O, PAS, myoadenylate deaminase, and myophosphorylase (20). Moreover, thin sections were evaluated with an electron microscope (EM 109 Zeiss) (20, 21).

To isolate genomic DNA, peripheral blood was taken from the proband and her family members. We used previously described primers (2) and Touch Down 64–54 PCR. The PCR products were purified by exonuclease I and alkaline phosphatase (Thermo Scientific) and were directly sequenced on both strands using an ABI 3130 Genetic analyzer with Big Dye Terminator chemistry. Sequence alignments were performed using the SeqScape v2.5 software (Applied Biosystems). After detecting the mutation in the proband, the genomic DNA of all family members was screened for variant segregation by sequencing exon 7 in the *STIM1* gene.

To estimate the mutation frequency, clinical presentation and genotype/phenotype correlation of Stormorken syndrome, we performed a comprehensive literature search in MEDLINE from 1985 until September 2017 and retrieved 11 studies reporting this condition. The key words utilized for the database-search were the following: “STIM1,” “Stormorken syndrome,” and “Tubular aggregate myopathy.” The collected data included the clinical phenotype, gender of all patients, age of onset, laboratory, and histological data and genotype. We then compared these features between two main groups of patients: those who harbored mutations within the CC-hand domain (*n* = 19) and those who harbored mutations within the EF-hand domain (*n* = 30; Tables 1, 2).

**Table 1:** Genotype/Phenotype of STIM1 patients in literature.

**References**	**Patients**	**Gender**	**Age of onset**	**Inherited /*de novo***	**STIM1 Mutations**	**Protein domain**	**Muscular weakness**	**Contractures**	**Walking**	**Miosis**	**Asplenia**	**Thrombocytopenia or platelets dysfunction**	**CK level (IU/L)**	**Hypocalcaemia**	**Muscle biopsy, TAM**
Harris et al. (4)	F1-II-1	M	39 y.o.	*De novo*	c.910C>T (p.R304W)	CC	+	+	Ambulant	+	+	+	1500-5000	+	TAM
	F2-II-1	F	14 months	*De novo*	c.242G>A (p.G81N)	EF1	+	+	Ambulant	+	+	+	1125	+	TAM
	F3-II-2	F	Congenital	*De novo*	c.262A>G (p.S88G)	EF1	+	+	Non-ambulant	+	+	+	823	+	Dystrophic
	F4-II-5	M	48 y.o.	Inherited	c.911G>A (p.R304Q)	CC	–	+	Ambulant	+	–	–	800	–	TAM
	F4-II-2	F	?	Inherited	c.911G>A (p.R304Q)	CC	–	–	Ambulant	–	–	–	225	–	?
	F4-III-5	F	18 y.o.	Inherited	c.911G>A (p.R304Q)	CC	–	+	Ambulant	–	–	+	113	+	?
Noury et al. (3)	Proband	F	Childhood	*De novo*	c.252T>A (p.D84E)	EF1	Proximal muscle weakness with lower limb predominance	+	Ambulant	–	+	–	880	+	TAM
Walter et al., (22)	I.1	M	Early Childhood	Unknown	c.242G>A (p.G81N)	EF1	Severe, upper and lower limbs, predominantly proximal	Neck, elbows, wrists, fingers, hips and Achilles tendon	Ambulant with cane since age 20, wheelchair since age 27	+	–	–	420	?	TAM
	II.1	M	Early Childhood	Inherited	c.242G>A (p.G81N)	EF1	Lower limbs proximal, upper limbs mild proximal	Mild in elbows, wrists, fingers and hip	Ambulant, hyperlordosis and fixed knee joints	–	?	?	500	?	Mainly in type I fibers, VMC and IC
Morin et al. (2)	F1-I-3	M	?	Unknown	c.910C>T (p.R304W)	CC	+/–	?	?	+	+	+	4412	+	TAM
	F1-II-2	M	?	Inherited	c.910C>T (p.R304W)	CC	+	?	?	+	–	+	769	+	TAM
	F2-II-2	F	?	Unknown	c.910C>T (p.R304W)	CC	+	?	?	+	+	+	919	+/-	?
	F2-III-1	F	?	Inherited	c.910C>T (p.R304W)	CC	-	?	?	+	–	+	491	?	?
Misceo et al. (6)	F1-II-2	F	4 y.o.	Unknown	c.910C>T (p.R304W)	CC	++	?	Ambulant	+	+	+	487-875	+	?
	F1-III-1	M	3.5 y.o.	Inherited	c.910C>T (p.R304W)	CC	++	?	Ambulant	+	+	+	2703-2814	+	TAM
	F2-II-3	M	26 y.o.	*De novo*	c.910C>T (p.R304W)	CC	+	?	?	+	+	+	820-1718	+	?
	F3-II-1	M	40 y.o.	*De novo*	c.910C>T (p.R304W)	CC	+/-	?	?	+	Splenectomy	–	4000-5000	–	TAM
	F4-II-8	F	Adolescence	Unknown	c.910C>T (p.R304W)	CC	+	?	?	+	+	+	327	?	?
	F4-III-I	F	Childhood	Inherited	c.910C>T (p.R304W)	CC	+	?	?	+	+	+	1923-4017	–	TAM
Nesin et al. (1)	F1-Case1	F	?	Unknown	c.910C>T (p.R304W)	CC	+	?	?	+	+	+	1668	?	?
	F2-Case2	M	?	Unknown	c.910C>T (p.R304W)	CC	+	?	?	+	+	+	3819	?	?
Markello et al., (23)	A-II-2	F	< 2 y.o.	Unknown	c.343A>T (p.I115F)	EF2	Predominantly proximal	?	Ambulant with cane	+	+	+	450	?	?
	A-III-1	M	Congenital	Inherited	c.343A>T (p.I115F)	EF2	Predominantly proximal	?	Ambulant	+	–	+	2451	?	?
	A-IV-1	M	Congenital	Inherited	c.343A>T (p.I115F)	EF2	?	?	?	–	?	+	751	?	?
Markello et al., (23)	B-II-2	F	1 year old	*De novo*	c.343A>T (p.I115F)	EF2	Difficult in climbing stairs, and walking long distances	?	?	–	?	+	668	?	?
	C-II-1	M	Congenital	*De novo*	c.910C>T (p.R304W)	CC	?	?	?	–	?	+	412	?	?
	D-1-1	M	Childhood	Unknown	c.910C>T (p.R304W)	CC	Predominantly proximal	?	?	–	?	?	3000-4000	?	?
	D-II-1	M	Congenital	Inherited	c.910C>T (p.R304W)	CC	?	?	?	–	?	+	350-500	?	?
Okuma et al. (24)	Proband	M	37 y.o.	Inherited	c.1450_145 1insGA (p.Ile484Arg fsX21)	CTID	+	+	Ambulant, lordotic	–	–	–	790	–	TAM
Hedberg et al., (25)	Patient 1	F	Early Childhood	*De novo*	c.326A>G (p.H109R)	EF2	Proximal muscle weakness in both upper and lower extremities	–	Ambulant, wide-based, drop foot	?	?	?	1300	?	TAM
	Patient 2	F	3 y.o.	*De novo*	c.326A>G (p.H109R)	EF2	Limb girdle	+	Ambulant, waddling gait	?	?	?	600-1200	?	TAM
	Patient 3	F	Early Childhood	*De novo*	c.343A>T (p.I115F)	EF2	Limb girdle	+	Ambulant	?	?	?	1200-1500	?	TAM
Bohm et al., (26)	F1 – I.1	F	Adolescence	Unknown	c.251A>G (p.D84G)	EF1	Lower limbs proximal	–	Ambulant	?	?	?	elevated	?	?
	F1-II.1	M	Childhood	Inherited	c.251A>G (p.D84G)	EF1	Lower limbs proximal, upper limbs mild and diffuse	Heels, wrists, fingers	Ambulant	?	?	?	8x	?	In type I and II fibers, VMC and IC
	F1-II.2	M	Childhood	Inherited	c.251A>G (p.D84G)	EF1	Lower limbs proximal	Heels, wrists, fingers	Ambulant	?	?	?	4x	?	?
	F2-II.2	F	Adolescence	Inherited	c.325C>A (p.H109N)	EF2	Lower limbs proximal	–	Ambulant	?	?	?	4x	?	In type I and II fibers, VMC and IC
	F2-II.10	M	Childhood	Inherited	c.325C>A (p.H109N)	EF2	Lower limbs proximal	Knees	Ambulant	?	?	?	5x	?	In type I and II fibers, VMC and IC
	F2-III.1	F	Childhood	Inherited	c.325C>A (p.H109N)	EF2	Lower limbs proximal, upper limbs mild and proximal	Heels	Ambulant	?	?	?	7x	?	Mainly in type I fibers, VMC and IC
	F3-I.2	M	Childhood	Unknown	c.326A>G (p.H109R)	EF2	Severe, upper and lower limbs, predominantly proximal	Neck, elobows, wrists, fingers	Non-ambulant	?	?	?	5x	?	Mainly in type I fibers, VMC and IC
Böhm et al., (26)	F3-II.2	M	Childhood	Inherited	c.326A>G (p.H109R)	EF2	Lower limbs proximal, upper limbs mild and proximal	Neck, heels	Ambulant	?	?	?	12x	?	In type I and II fibers, VMC and IC
	F4-I.2	M	Asymptomatic	Unknown	c.216C>G (p.H72Q)	EF1	–	–	Ambulant	?	?	?	5x	?	In type I and II fibers, VMC and IC
	F4-II.1	M	Asymptomatic	Inherited	c.216C>G (p.H72Q)	EF1	–	–	Ambulant	?	?	?	27x	?	In type I and II fibers, VMC and IC
	F4-II.2	M	Asymptomatic	Inherited	c.216C>G (p.H72Q)	EF1	–	–	Ambulant	?	?	?	9x	?	In type I and II fibers, VMC and IC
Böhm et al., (27)	F1-II.1	M	Childhood	*De novo*	c.239A>C (p.N80T)	EF1	Proximal, lower limbs	–	Ambulant	?	?	?	10x	?	TAs (mainly type II), VCM
	F2-II.6	M	Adulthood	Unknown	c.239A>C (p.N80T)	EF1	Proximal, predominantly lower limbs	Heels	Ambulant, foot drop, unable to walk on heels	?	?	?	6x	?	TAs, IC
	F3-II.1	F	Childhood	*De novo*	c.286C>G (p.L96V)	EF1	Lower limbs	–	Ambulant	?	?	?	8x	?	TAs mainly (type II), IC
	F4-II.2	M	Childhood	Inherited	c.322T>A (p.F108I)	EF2	Predominantly proximal	Heels	Ambulant with support, unable to walk on heels	?	?	?	4x	?	TAs,n.a.
Böhm et al., (27)	F4-II.3	F	Childhood	Inherited	c.322T>A (p.F108I)	EF2	Predominantly proximal	Heels	Ambulant with support, unable to walk on heels	?	?	?	?	?	TAs, IB and IC
	F5-II.2	F	40 y.o.	Unknown	c.322T>A (p.F108I)	EF2	–	–	Ambulant	?	?	?	Normal	?	TAs, n.a.
	F6-II.1	M	Childhood	Unknown	c.325C>A (p.H109N)	EF2	–	–	Ambulant	?	?	?	15x	?	TAs, type I fibre predominance, VCM and IC

*y.o., years old; F, female; M, male; CC, coiled-coil domain; EF, EF-Hand domain; IU/L, International Units Per Litre; CK, Creatine Kinase; TAM, tubular aggregate myopathy; CTID, C-terminal inhibitory domain; VMC, vesicular membrane collection; IC, tubular aggregates with empty or moderately dense flocculent material; TAs, tubular aggregates (Classification, Ib, tubular aggregates with central tubule; Ic, tubular aggregates with empty or moderately dense flocculent material); n.a., not assessed*.

**Table 2:** Clinical features of STIM1 mutations CC and EF.

	**CC (*n* = 19)**	**EF (*n* = 30)**
Gender, M (%)	58	53
**Age of onset (%)**		
Asymptomatic	0	3 (10)
Congenital	2 (10)	3 (10)
Childhood/Adolescence	6 (31)	22 (73)
Adulthood	4 (21)	2 (7)
Myalgia, yes (%)	8 (42)	11 (37)
Muscular weakness, yes (%)	13 (68)	24 (80)
Contractures, yes (%)	3 (16)	16 (53)
Ambulant, yes (%)	6 (32)	25 (83)
Miosis, yes (%)	14 (73)	5 (17)
Ophtalmoplegia, yes (%)	2 (10)	11 (37)
Thrombocytopenia/platelets dysfunction, yes (%)	15 (79)	6 (20)
Asplenia/hyposplenia, yes (%)	10 (53)	4 (13)
Cardiac dysfunction, yes (%)	0 (0)	3 (10)
Respiratory dysfunction, yes (%)	0 (0)	4 (13)
Hypocalcemia, yes (%)	8 (42)	3 (10)

## Results

### Clinical findings: history and clinical examination

A 21-year-old female of Italian origin was referred to our Neuromuscular Center because of chronic CK elevation that ranged from 439 to 2350 UI/l and mild symptoms. She only complained of episodes of diffuse myalgia during fever episodes. Because of these symptoms, she was admitted to the emergency department three times over the last three years, during which she underwent blood tests, urinalyses, chest radiographs, arterial blood gas measurements, electrocardiography, and abdominal and cardiac ultrasounds. High CK serum levels, low blood platelet counts, a low calcium serum concentration (corrected for albumin level) and a reduced spleen size were detected.

Her past medical history revealed several associated clinical conditions, including the following: congenital bilateral miosis without eye pain, an increased ocular tone or other signs of retinopathy; a diagnosis of idiopathic thrombocytopenic purpura (ITP) when she was 6 years old with several episodes of mucosal bleeding, such as epistaxis and menorrhagia. A diagnosis of dyslexia was made in the same year; ten years later, the patient was diagnosed with an episode of autoimmune thyroiditis associated with antithyroglobulin antibodies. Finally, in the last year, a functional platelet defect characterized by impaired platelet activation and secretion after stimulation with ADP, collagen and TRAP (Thrombin receptor-activating peptides) was discovered.

**Figure 1:**
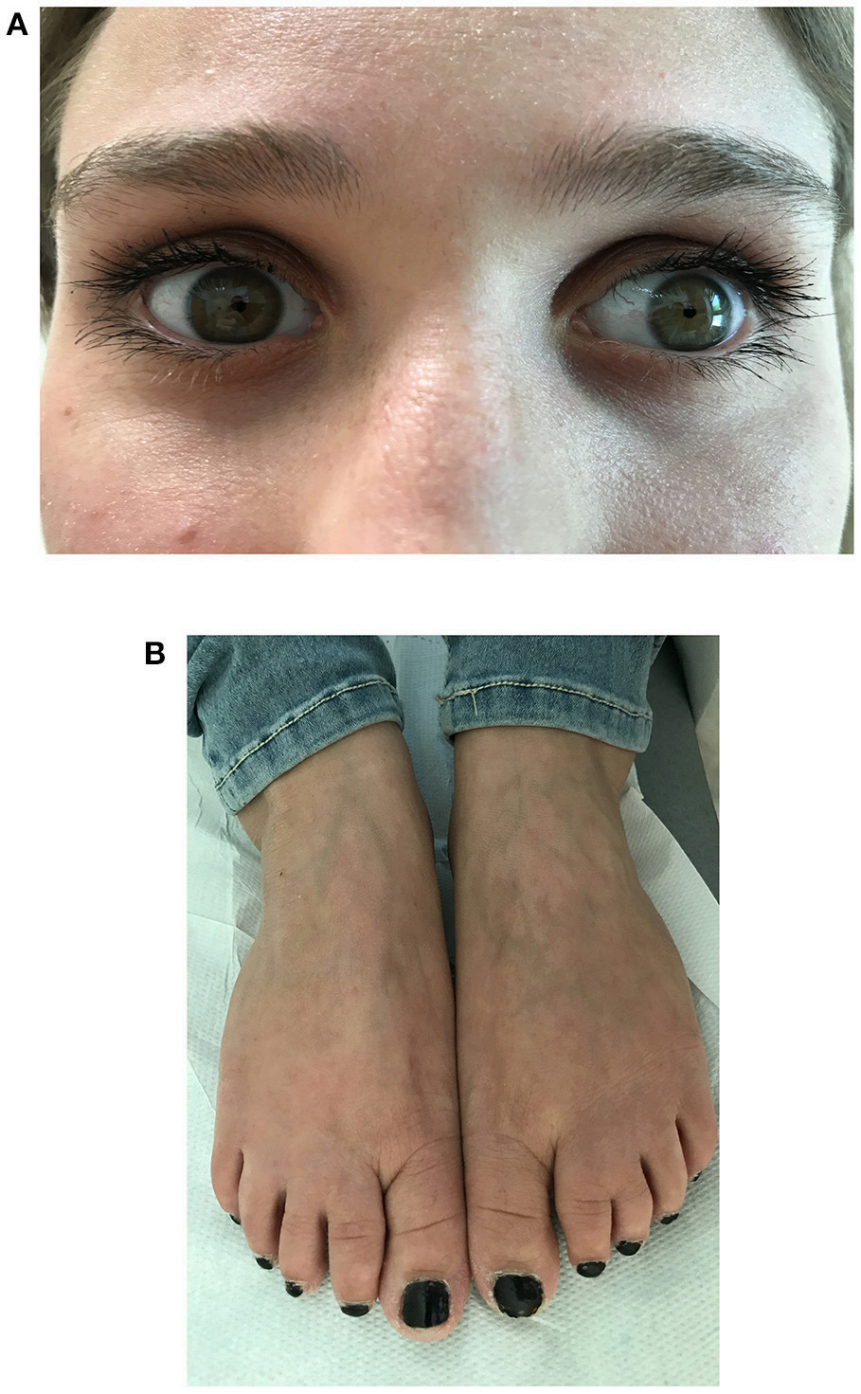
Morphological and ophthalmological findings in a 21-year-old Italian female with Stormorken syndrome. **(A)** Close-up photo of the patient's eyes showing bilateral and symmetric miosis. **(B)** Photo of the patient's feet showing partial syndactyly of the second and third toes.

A clinical examination revealed a short stature (148 cm) without dysmorphic signs. The patient appeared alert and oriented to person, place, and time. The clinical examination of the cranial nerves revealed bilateral, symmetric miosis (Figure 1A) and a poor response to a light stimulus without diplopia or ptosis. Examination of the ocular extrinsic musculature revealed no defects. She presented with very mild symmetrical muscle weakness with predominant impairment of the proximal lower limbs (MRC score 5-/5) and an even milder involvement of the axial muscles and upper limbs. No other abnormalities of motor or sensory functions were detected except for a slight postural tremor in the upper arms when the arms were outstretched. No abnormalities in gait, posture or coordination were detected. She was independently ambulant, even if she reported exercise intolerance. Partial syndactyly of the second and third toes (Figure 1B) was detected when the lower limbs were examined.

Blood tests revealed elevated CK serum levels (898 U/L), low platelet counts (87000/mm3), hypocalcemia (2.03 mmol/L) with normal phosphatemia and a serum PTH concentration of 9.2 pg/mL, which was inappropriately low given her low calcium level. All of the other laboratory findings were normal.

Electroneurography did not reveal alterations in the amplitude of the compound muscle action potentials or sensory nerve action potentials. Electromyography did not reveal pathological findings.

**Figure 2:**
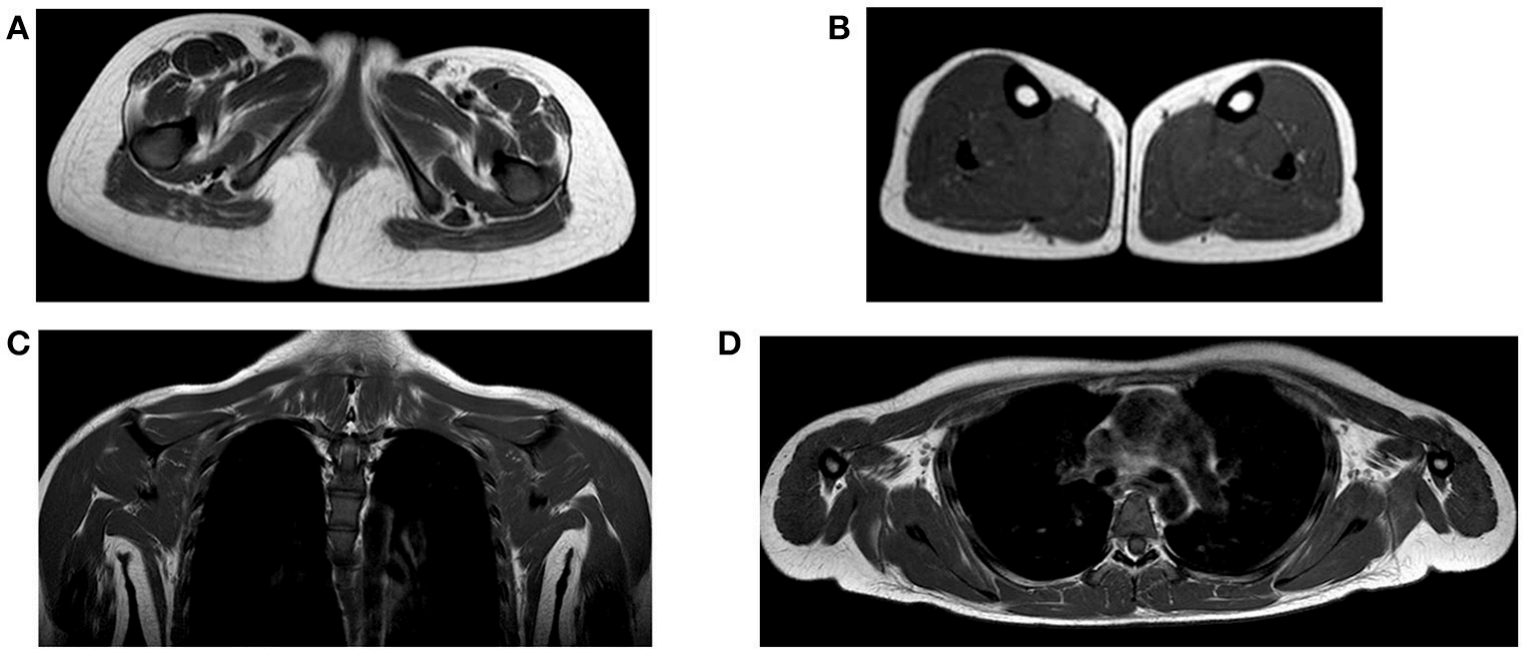
**(A)** Axial T1 image at the pelvis showing atrophy and moderate fatty replacement involving the gluteus maximus, particularly on the right side (black arrow); **(B)** axial T1 MR image at the level of the calf showing the relative sparing of the muscles with only moderate involvement of the gastrocnemii lateralis (dotted white arrow); **(C,D)** coronal and axial T1 MR images at the level of the cingular girdle showing mild fatty infiltration in the subscapularis muscles.

Brain and brainstem MRI (without contrast medium) showed no abnormal focal areas of altered signal intensity in the cerebral hemispheres, brainstem, or cerebellum. Lower limb MR imaging revealed a mild fatty replacement within the gluteus maximus muscles, especially in the right gluteus maximus (Figure 2A). In the leg, the gastrocnemius lateralis was slightly affected by fatty replacement (Figure 2B). There was sparing of the tibialis anterior and posterior muscles (Figure 2B). T1-weighted MR imaging of the upper limb and cingular girdle demonstrated some alterations that were characterized by mild fatty infiltration in the subscapularis and pectoralis major (Figures 2 C,D). The masticatory muscles (temporal, masseter and lateral pterygoid) were normal. In the upper limbs, the triceps and biceps brachii were spared. No bright signal was observed on the STIR sequence, suggesting the absence of areas with inflammatory edema. Compared to other reported cases, only mild changes were described. Moreover, in STIM1 disease, a peculiar involvement of the flexor halluces longus, which was not present in this case, has been reported.

**Figure 3:**
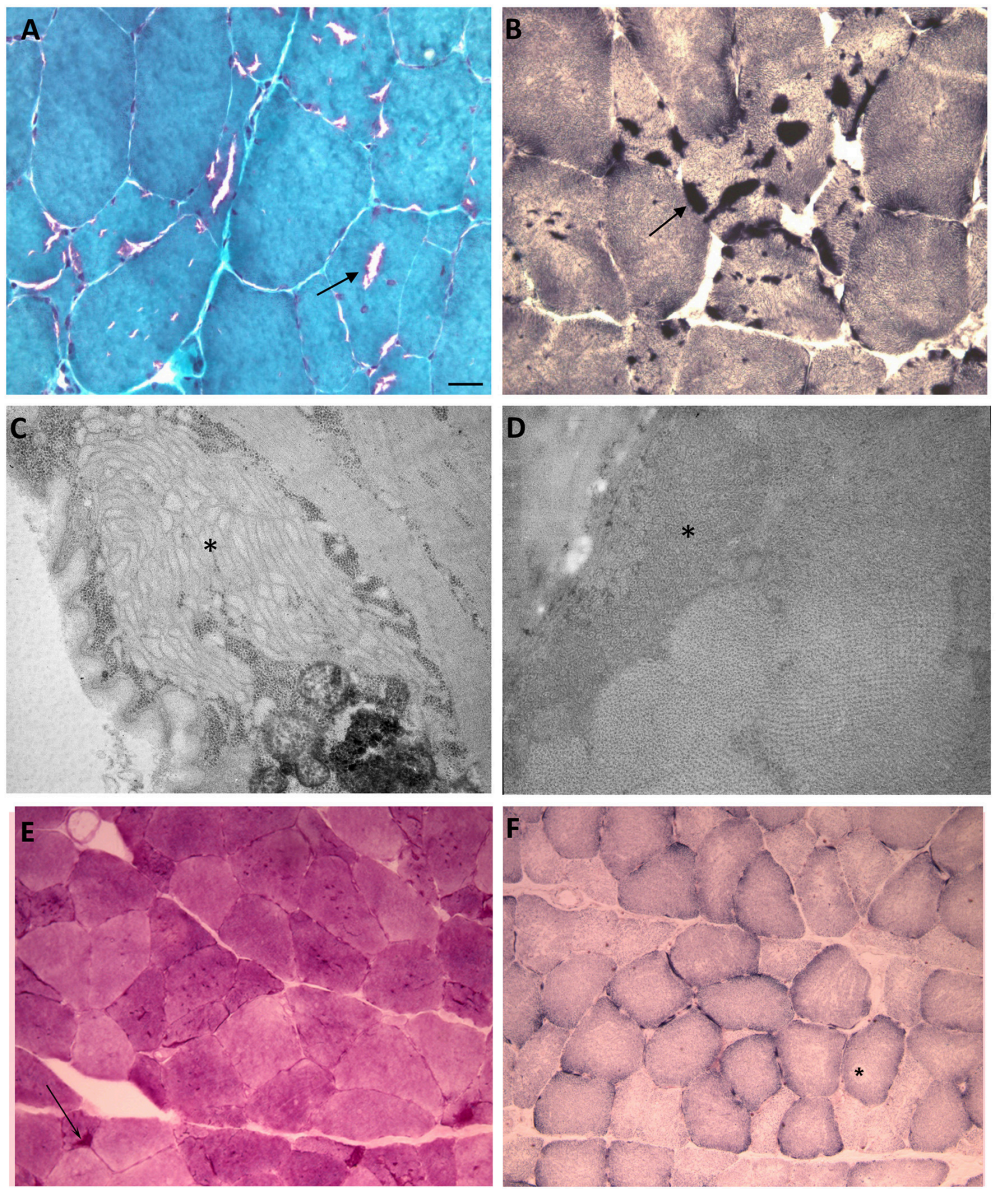
Histological, histochemical and ultrastructural findings. **(A)** A Modified Gomori Trichrome stain (40X) showing tubular aggregates and a lack of tubular aggregates. **(B)** NADH (40X) reaction showing many cytoplasmic dark stained areas. **(C,D)** (**C**: 12000X) (**D**: 20000X) EM. Subsarcolemmal evidence of tubular aggregates (asterisks). **(E)** small accumulations of PAS positive material was detected in some fibers. **(F)** SDH staining showed no accumulations even in the described vacuolated areas. Scale bar: **(A,B)** 12.5 μm **(C)** 0.42 μm **(D)** 0.25 μm **(E, F)** 25 μm.

A histological examination of the biopsy of the left biceps (Figures 3A–F) revealed the presence of diameter variations of type 1 and 2 fibers. We also detected multiple basophilic cytoplasmic inclusions in many muscle fibers that were consistent with tubular aggregates (TA; Figure 3A). No signs of necrosis or inflammation were observed. Slightly augmented internalized nuclei, few fiber splittings and type 2 fiber atrophy were observed. A histochemical examination including the NADH reaction showed many cytoplasmic dark stained areas (Figure 3B). The areas positive for NDAH were negative for SDH, supporting an endoplasmic reticulum origin (Figure 3F). Moreover, the histochemical analysis detected small accumulation of PAS-positive material (Figure 3E) In conclusion, the histopathological data obtained from examining the biceps sample revealed significant pathological muscular involvement indicative of tubular aggregate myopathy.

The electron microscopy analysis revealed the presence of tubular aggregates constituting single-walled (250 nm diameter) membrane tubules (Figures 3C,D).

**Figure 4:**
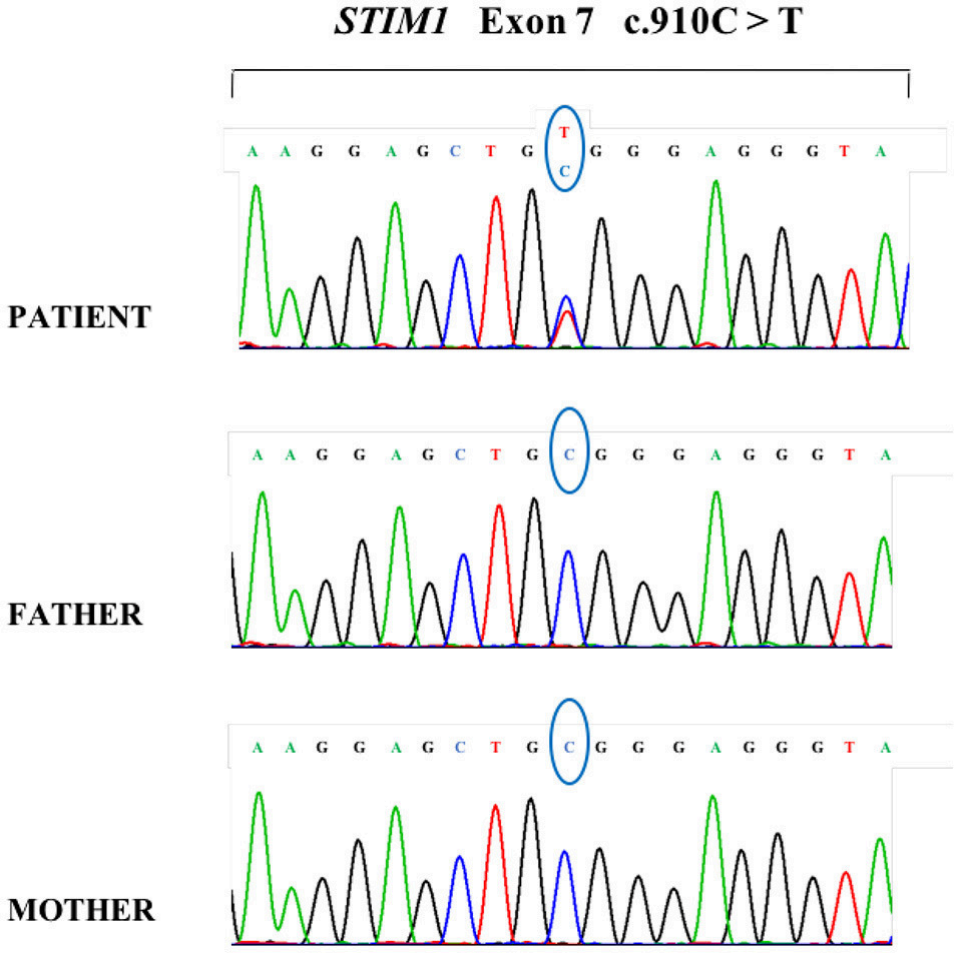
DNA sequencing analysis of the patient and her parents demonstrating a *de novo* heterozygous transition in the patient with the common c.910C>T mutation in *STIM1*.

*STIM1* gene analysis in our patient using Sanger direct sequencing showed a *de novo* heterozygous missense mutation, a c.910C > T transition (NM_003156.3; rs483352867), which caused a p.R304W amino acid substitution in the protein and affected a highly conserved cytoplasmic CC domain (Figure 4). This protein alteration, which has already been reported in the literature in patients with Stormorken syndrome (6), impaired the conformation of the STIM1 inhibitory helix by unlocking the inhibitory state of STIM1 (2) and was able to stimulate the CRAC channel. This mutation was absent in the unaffected relatives (Figure 4).

### Correlation between the genotype and phenotype in stormorken syndrome: a review of the literature

We conducted a review of published STIM1 (dominant) gain of function mutations (*n* = 50). Patients were grouped according to their STIM1 genotype: 19/50 harbored mutations within the CC-hand domain and 30/50 patients harbored mutations within the EF-hand domain (Table 1). One patient harbored a mutation within the CTID domain (24). All reported classic Stormorken patients harbored a common mutation in p.R304W in a coiled coil domain. Moreover, when these genotypic groups were compared, CC-hand domain mutations were found to be associated with a more complete extra-muscular involvement (presence of miosis, thrombocytopenia/platelet dysfunction, asplenia or hyposplenia and hypocalcemia) than those harboring EF-hand domain mutations. These data suggest a genotype-phenotype correlation between mutations within the coiled coil domain that is associated with complete Stormorken syndrome, whereas mutations outside of this region more often present with an incomplete phenotype.

## Discussion

Stormorken syndrome, the only recognized syndrome combining myopathy and congenital hypo/asplenia, was reported in 1985 by Stormorken et al. in a family from Norway that had an autosomal dominant transmission. Stormorken syndrome is a rare condition in which a combination of hypocalcemia, hematological abnormalities (including asplenia, anemia, thrombocytopenia, or thrombocytosis), TAM and other features, such as short stature, miosis, migraine, dyslexia, and ichthyosis, may be observed (5). Recent reports have described that a wide range of STIM1 mutations are the cause of Stormorken syndrome: the most common mutation, p.R304W, involves the coiled coil domain and appears to lead to a gain of function of the STIM1 protein (1, 2, 6).

Our patient who was of Italian origin presented with the clinical features of Stormorken syndrome, including myopathy, miosis, bleeding tendency, thrombocytopenia, hyposplenia, and dyslexia.

Muscular involvement in Stormorken syndrome consists of a gradually progressive muscle weakness, primarily of the proximal lower limb, in association with exercise intolerance, joint contractures, ophthalmoparesis without diplopia or ptosis, and elevated CK levels (15). Our patient showed a phenotype consistent with the previously described phenotype and presented with increased CK, mild muscle weakness, exercise intolerance and sporadic myalgia. In general, the CK serum levels reported in the literature show very high variability among cases, independent of the severity of the symptoms or the localization of the mutation within the STIM1 gene. It may be possible that other unrecognized genetic and epigenetic factors can act as modifiers of the clinical phenotype by altering calcium homeostasis, stabilizing muscular cell membranes or influencing cellular stress-response pathways. Further research should be performed to clarify this high variability.

Muscle imaging was performed in five patients with STIM1-associated TAM (28). In the lower limb, fatty substitution of the obturator and gluteal muscles was found in early disease stages. Similar findings were found in the sartorius muscle.

In later stages, general alterations of the anterior and posterior thigh were found, with relative preservation of the adductor longus, gracilis, and the short head of the biceps femoris. In the lower leg, alterations of the posterior compartment (gastrocnemii and soleus) were present. In the upper limb, the subscapularis muscle was consistently involved and the trapezius was preserved. However, only mild changes were observed in our case.

Muscle biopsy agreed with the histological and electron microscopy findings of TAM.

Sanger analysis of the patient's genome revealed the common p.R304W mutation.

Previous functional analyses suggested that this mutation causes constitutive STIM1 activation, with a consequent dysregulation of intracellular Ca^2+^ homeostasis and an increase in the basal Ca^2+^ levels in patient's derived fibroblasts and STIM1-R304W overexpressing HEK 293T cells (2). Therefore, the administration of SOCE inhibitors, such as resveratrol, could be a possible therapeutic approach (29). Resveratrol is a natural phytoalexin that is used as a nutrition supplement due to its health-promoting benefits and is an inhibitor of store-operated calcium entry (SOCE) (29). In fact, it has been demonstrated that resveratrol inhibits the ERK1/2 activation triggered by Ca2^+^ store depletion. Since resveratrol is already used in humans, it can be transferred to the clinic. Indeed, further knowledge of the molecular mechanism underlying this inhibition will allow the effective design of therapies that include resveratrol or related stilbenoids (29).

In a patient with hypocalcemia, the use of calcitriol was shown to have a significantly ameliorated neuromuscular function (30), but these findings were not investigated further.

Mutations in STIM1 are believed to cause either TAM or Stormorken syndrome in correlation with the domain that the mutation affects. To clarify the correlation between the STIM1 genotype and phenotype, we reported comprehensive clinical and genetic features from 50 patients described in the literature with STIM1 dominant gain of function mutations in the EF-hand or CC domains. Our findings suggest that extra-muscular features, such as hypocalcemia, thrombocytopenia, platelet defect and hyposplenia or asplenia, are related to mutations within the coiled domain of the STIM1 protein. Moreover, the neuromuscular phenotype was substantially different according to which STIM1 protein domain was affected; subjects with EF-region domain mutations presented with a more severe neuromuscular dysfunction that was characterized by progressive hyposthenia and contractures and earlier onset than patients harboring mutations in the CC region domain, in which neither weakness nor contractures was reported. In fact, mutations in the EF-hands constitutively activate STIM1, leading to permanent, store-independent calcium entry. By contrast, mutations in the STIM1 CC domains confer a gain of function that is only revealed by store depletion. Therefore, tissues relying on store-operated calcium entry for differentiation and function might be preferentially affected by this mutation (1). This mechanistic difference might explain the genotype-phenotype correlation.

Our study is the first report of an Italian patient with Stormorken syndrome and confirms the association between the most common *STIM1* gain of function mutation (c.910C>T, p.R304W) within the CC domain and the majority of the clinical features already described in Stormorken syndrome.

Our data strengthen this genotype/phenotype correlation between the *STIM1* p.R304W mutation and points to the possibility of directly investigating this mutation in the presence of a typical phenotype.

## Author contributions

OB: data acquisition, manuscript writing. DP: molecular analysis. SCos: data acquisition, review of literature, manuscript writing. AG and FM: data acquisition. AA: haemathological clinical data acquisition. CC: MRI analysis. GF: muscle pathology data. PC: histopathological experiments. MM: muscle data interpretation and analysis. NB and GC: study support and manuscript revision. SCor: data acquisition, manuscript writing.

### Conflict of interest statement

The authors declare that the research was conducted in the absence of any commercial or financial relationships that could be construed as a potential conflict of interest.
